# Phenolic Profile and Antioxidant Activity of Italian Monovarietal Extra Virgin Olive Oils

**DOI:** 10.3390/antiox8060161

**Published:** 2019-06-05

**Authors:** Carmine Negro, Alessio Aprile, Andrea Luvisi, Francesca Nicolì, Eliana Nutricati, Marzia Vergine, Antonio Miceli, Federica Blando, Erika Sabella, Luigi De Bellis

**Affiliations:** 1 Department of Biological and Environmental Sciences and Technologies (DiSTeBA), Salento University, Via Prov. le Lecce-Monteroni, 73100 Lecce, Italy; carmine.negro@unisalento.it (C.N.); alessio.aprile@unisalento.it (A.A.); andrea.luvisi@unisalento.it (A.L.); francesca.nicoli@unisalento.it (F.N.); eliana.nutricati@unisalento.it (E.N.); marzia.vergine@unisalento.it (M.V.); antonio.miceli@unisalento.it (A.M.); luigi.debellis@unisalento.it (L.D.B.); 2 Institute of Sciences of Food Production (ISPA), National Research Council (CNR), Research Unit of Lecce, Via Prov. le Lecce-Monteroni, 73100 Lecce, Italy; federica.blando@ispa.cnr.it

**Keywords:** monovarietal extra virgin olive oil, phenolic compounds, oleuropein, oleocanthal, antioxidant activity, DPPH, ORAC, high-performance liquid chromatography time-of-flight mass spectrometry (HPLC/MS/TOF)

## Abstract

In the last years, the interest in Italian monovarietal oils has increased due to their specific organoleptic qualities. Extra virgin olive oils (EVOOs) are rich in phenolic compounds, secondary metabolites well known and studied for their nutraceutical properties. However, among EVOOs, there is great variability in phenolic composition due to the origin, the production technique, and mainly, the genotype. The aim of this work was to evaluate the different phenolic profiles and the antioxidant activities of monovarietal oils. The results confirm this variability. In fact, the overall content of oleuropein varies up to four times between the different genotypes (from 33.80 to 152.32 mg/kg oil), while the oleocanthal content is significant only in two oils. The antioxidant activity, determined with 2,2-Diphenyl-1-picrylhydrazyl (DPPH) and oxygen radical absorbance capacity (ORAC) assays, is correlated with the content of total phenolic substances, with half maximal inhibitory concentration (IC_50_) values for the DPPH test ranging from 160 to 91 mg of oil, while the ORAC test shows values between 5.45 and 8.03 μmol Trolox equivalent (TE)/g oil.

## 1. Introduction

Extra virgin olive oil (EVOO) is the unique oil obtained by mechanical cold processes from the fruits of the olive. EVOO mainly consists of triglycerides (about 98%), and among them, oleic acid represents the main component (about 50 to 80%). Linoleic acid (from 3.5% up to 20%) and small amounts of linolenic acid (about 1%) are usually found in EVOO too. In the remaining 2%, there are several compounds such as phytosterols, vitamin E, triterpenes, and aliphatic alcohols, as well as a series of phenolic compounds that characterize the quality of the EVOO [1].

The phenolic compounds typically present in EVOO are mainly derivatives of hydroxytyrosol and tyrosol and are classified as secoiridoids. There are also small quantities of lignans, pinoresinol and acetoxypinoresinol being the most common. Among phenolic acids, vanillic, coumaric, and protocatechuic acid can be found in EVOO. Finally, there are some flavonoids, among which luteolin and apigenin are the most important [2,3,4,5,6,7,8].

Phenolic compounds have been extensively studied for their nutraceutical properties. In particular, the antioxidant activity has been evaluated with different methodologies, both in vitro and in vivo [5,6,7,8,9,10,11,12]; moreover, the oxidation state of plasma in humans decreases after the intake of EVOO with a high content of polyphenols [13]. In patients with mild dyslipidemia, the intake of EVOO decreases the production of thromboxane B2 and the excretion of isoprostane, two markers of coronary heart disease (CHD) risk [14]. In patients with mild hypertension, the intake of extra virgin olive oil improves the oxidation state of plasmatic low-density lipoproteins (LDL), decreases their peroxidation, increases the reduced glutathione, and decreases hypertension [15]. In a trial with 200 volunteers, the intake of extra virgin olive oil led to an increase in high-density lipoproteins (HDL) and a simultaneous reduction of in oxidation state of plasma [16]. It was also observed that the use of EVOO can reduce (up to 30%) the presence of markers associated with oxidized DNA in menopausal women [17], while those who feed themselves with extra virgin olive oil, instead of sophisticated oil or corn oil, have a reduction in plasma inflammation markers (thromboxane B2 and leukotriene B4) [18]. A different trial demonstrated that the use of EVOO reduces the risk of osteoporotic fractures [19]. Finally, the pharmacological properties and action’s mechanisms of oleocanthal (decarboxymethyl ligstroside aglycone) were recently summarized by Francisco et al. [20]. With all this evidence, the US Food and Drug Administration has authorized the claim against cardiovascular diseases [21] and the European regulation 432/2012 allows EVOOs with a polyphenol content higher than 5 mg/20 g to claim “Olive oil polyphenols contribute to the protection of blood lipids from oxidative stress” [22].

A great variability in the presence and levels of phenolic compounds among EVOOs exists due to the olive genotype, cultivation area of trees, and the oil extraction technique. This variability greatly affects the organoleptic and nutraceutical differences between EVOOs [23,24,25,26,27].

The aim of this work was to evaluate the different phenolic compounds profiles of mono-varietal EVOOs obtained from eight different genotypes grown in the same climatic area and to estimate their antioxidant activity.

## 2. Materials and Methods

### 2.1. Plant Material and Oil Production

Oils from eight genotypes/cultivars (Table 1) were extracted from olives of trees grown in an identical climate/environment area: the province of Lecce, Apulia, Italy (40°21’17.32” N, 18°10’20.78” E). To limit the variation in phenolic content due to the ripening stage, the olives were harvested when they reached a ripening index (Jaén index) equal to 3 [25,28] and then processed with a mini-laboratory crusher. The oil was stored in amber-colored bottles. Immediately after oil production, the bottles were closed and kept in an environment protected from sunlight. Oil analyses were completed within a month after olive oil production. The oils were analyzed and classified as EVOO according to EU laws (data not shown).

### 2.2. Chemicals

Water, methanol, acetonitrile, and hexane, were high-performance liquid chromatography (HPLC)-grade and were provided by Sigma Aldrich (Milan, Italy), as well as the 6-Hydroxy-2,5,7,8-tetramethylchromane-2-carboxylic acid (Trolox), 2,2-Diphenyl-1-picrylhydrazyl (DPPH), hydroxytyrosol, oleuropein, pinoresinol, luteolin, and apigenin analytical standard grade.

### 2.3. Phenolic Compounds: Extraction, Identification, and Quantification

The phenolic compounds were extracted by placing 5 g of oil into a 50 ml tube. For a better oil fluidification, 2 ml of hexane were added and subsequently, 5 ml of methanol: water 80:20 *v*/*v*. The solution was vortexed for 10 min. The emulsion was subjected to centrifugation for 20 min at 5500× *g* at 4 °C to separate the two phases. The alcoholic extract was recovered, and this procedure was repeated three times. Finally, the alcoholic extract was evaporated in cold and reduced pressure conditions. The dried extract was resuspended in 1 ml of 80% methanol.

The total phenolic content was determined using the spectrophotometric Folin–Ciocalteu method [29]. Data were expressed as mg of gallic acid equivalent (GAE) per kg of EVOO.

The phenolic identification was performed using the Agilent 1200 Liquid Chromatography System (Agilent Technologies, Palo Alto, CA, USA) equipped with a standard autosampler and Agilent column Zorbax extended C_18_ 50 × 2.1 mm, 1.8 μ. The separation was carried out at 30 °C with a gradient elution program at a flow rate of 0.4 mL/min. The mobile phases consisted of water plus 0.1% formic acid (A) and acetonitrile (B). The following multistep linear gradient was applied: 0 min, 10% B; 10 min, 25% B; 14 min, 50% B; 20 min, 80% B; 20 min 90% B. The injection volume in the HPLC system was 5 μL. The HPLC system was coupled to an Agilent diode array detector (DAD) (λ detection was 280 and 330 nm) and Agilent 6320 Time-of-Flight (TOF) mass spectrometer equipped with a dual electro spray interface (ESI) (Agilent Technologies) operating in negative ion mode. Detection was carried out within a mass range of 50–1700 *m*/*z*. Accurate mass measurements of each peak from the total ion chromatograms (TICs) were obtained by means of an Isocratic Pump (Agilent G1310B, company, Santa Clara, CA, USA) using a dual nebulizer ESI source that introduces a low flow (20 μL/min) of a calibration solution that contains the internal reference masses at *m/z* 112.9856, 301.9981, 601.9790, 1033.9881, in negative ion mode. The accurate mass data of the molecular ions were processed through the software Mass Hunter (Agilent Technologies, Santa Clara, CA, USA). The quantification of phenolic compounds was achieved using calibration curves of authentic chemical standards: hydroxytyrosol, oleuropein, pinoresinol, luteolin, and apigenin.

### 2.4. Antioxidant Activity

Antioxidant activity was evaluated using a 2,2-Diphenyl-1-picrylhydrazyl (DPPH) test as reported by Bondet et al. [30] and determined as half maximal inhibitory concentration, IC_50_ per mg of oil, and an oxygen radical absorbance capacity (ORAC) test, as reported by Ou et al. [31] and expressed as µmol Trolox Equivalent (TE)/g oil.

### 2.5. Statistical Analysis

All data were reported as the mean ± standard deviation (SD), with at least three replications for each olive oil. Statistical evaluation was conducted by Duncan’s test to discriminate among the mean values. To evaluate the correlation between antioxidant activity and total phenolic content (TPC) and between antioxidant activity and the single compounds, the Pearson correlation was calculated. Moreover, to provide a concise but comprehensive presentation of chemical compounds in relation to the different EVOOs, a heatmap was drawn [32].

## 3. Results

The total phenolic content (TPC) of eight monovarietal oils was expressed as gallic acid equivalents (Table 2). TPC ranged between 138 and 278 mg GAE/ kg, in the Spina and Ogliarola di Lecce cultivars, respectively. This result highlighted a great difference in the oil phenolic compounds content, suggesting that the genotype may be responsible for about 50% of this parameter.

**Figure 1:**
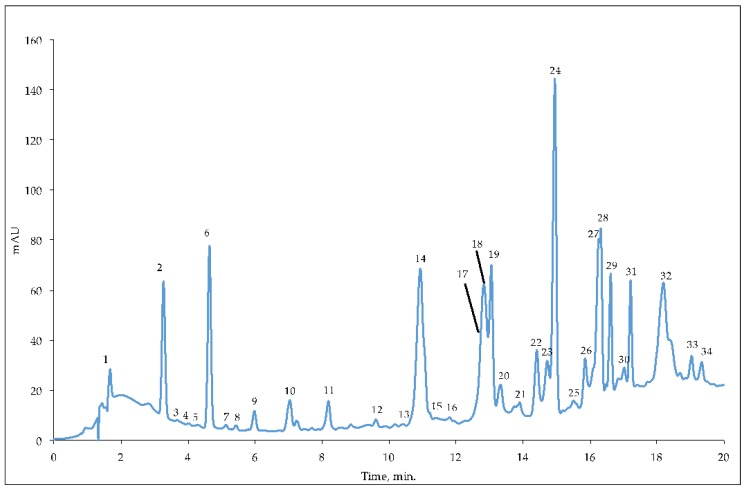
A representative high-performance liquid chromatography-diode array detection (HPLC / DAD) (*λ* = 280 nm) separation of the phenolic compounds present in the Oliva Grossa extra virgin olive oil. For the identification of the peaks and relative compounds, see Table 3.

As far as the identification and characterization of phenolic compounds in EVOO extracts is concerned, 30 compounds, already reported in other EVOO extracts [23,24,25,26,27], were identified, although these were not always present in all examined oils. Figure 1 displays a representative chromatogram of the phenolic extract obtained from the monovarietal oil of the Oliva Grossa cultivar (the chromatograms related to EVOO extracts from the other cultivars are essentially similar), while Table 3 shows the results of the qualitative analysis obtained by high-performance liquid chromatography time-of-flight mass spectrometry (HPLC/MS-TOF).

Hydroxytyrosol (peak 2, *m*/*z* 153.0563), hydroxytyrosol acetate (peak 4, *m*/*z* 195.0647), vanillic acid (peak 5, *m*/*z* 167.0349), tyrosol (peak 7, *m*/*z* 137.0610), coumaric acid (peak 8, *m*/*z* 163.0398), and ferulic acid (peak 13, *m*/*z* 193.0507) represent the class of simple phenolics.

Twenty secoiridoid molecules were identified in the extracts of the different EVOOs: deoxyelenoic acid isomer 1 and 2 (peak 3 and 11 *m*/*z* 225.0783), elenoic acid isomer 1, 2, and 3 (peak 6, 10 and 12, *m*/*z* 241.0731), decarboxymethyl oleuropein aglycone (peak 14, *m*/*z* 319.1313), decarboxymethyl 10 hydroxy oleuropein aglycone (peak 15, *m*/*z* 335.1163), oleuropein aglycone (peaks 16, 19, 24 and 25, *m*/*z* 377.1269), decarboxymethyl ligstroside aglycone (peak 18, *m*/*z* 303.1360), hydroxymethyl decarboxymethyl ligstroside aglycone (peak 20, *m*/*z* 333.1360), ligstroside isomer 1, 2, 3 and 4 (peak 21, 26, 29 and 30, *m*/*z* 361.1321), methyloleuropein aglycone (peak 27, *m*/*z* 391.1408), hydroxy oleuropein aglycone (peak 31, *m*/*z* 393.1205). Two lignans were also identified, pinoresinol (peak 17, *m*/*z* 357.1338) and acetoxypinoresinol (peak 23, *m*/*z* 415.1404), as well as two flavonoids, luteolin (peak 22, *m*/*z* 285.0432) and apigenin (peak 28, *m*/*z* 269.0459). Although the identified compounds have already been described in olive oils, the interesting point is the considerable difference in the levels of the different chemical compounds between the eight analyzed EVOOs, as shown in Table 4.

The main compound in the Colozzese and Barone di Monteprofico oils was oleuropein aglycone isomer 3, with 93.7 and 63.6 mg/kg, respectively, which is also present in a high concentration in Oliva Grossa EVOO. In Cellina di Nardò and Oliva Grossa, the dominant compound was decarboxymethyl oleuropein aglycone, with 73.6 and 124.4 mg/kg oil, respectively. Lastly, in Cornola, Ogliarola di Lecce, Oriella, and Spina, hydroxyoleuropein aglycone was the most represented compound. Among the other compounds, the content of decarboxymethyl ligstroside aglycone (oleocanthal) in the Colozzese and Oliva Grossa EVOOs, is especially meaningful, with values of 75.4 and 103.4 mg/kg, respectively. The amount of hydroxytyrosol was very variable. In particular, Colozzese contained about 16.1 mg/kg oil, while the other genotypes had lower values that ranged between 0.2 and 8.8 mg/kg oil. The luteolin and apigenin flavonoids were present in all EVOOs, with values ranging between 2.2 and 16.7 mg/kg (luteolin), respectively, in the Cornola and Oliva Grossa oils, and between 0.2 and 9.2 mg/kg (apigenin) in the Ogliarola di Lecce, Cellina di Nardò, and Cornola oils. High contents of lignans were observed in Barone di Monteprofico and Oliva Grossa EVOOs; the amounts of pinoresinol were 18.6 and 21.9 mg/kg oil, respectively. In contrast, Cellina di Nardò showed low levels of pinoresinol (1.4 mg/kg oil), whereas the mean value across all oils was 3.2 ± 2.1 mg/kg oil.

The antioxidant activity results are reported in Table 5. Relative to the DPPH test, the radical scavenging IC_50_ ranged between a best value of 91 mg (Oliva Grossa) and the worst of 160 mg (Spina). The mean IC_50_ was 102 mg. The antioxidant activity values measured by ORAC assay ranged between 5.45 µmol Trolox equivalent (TE)/g oil (Spina) and 8.03 µmol TE/g oil (Oliva Grossa). The mean value was 7.14 µmol TE/g oil.

**Figure 2:**
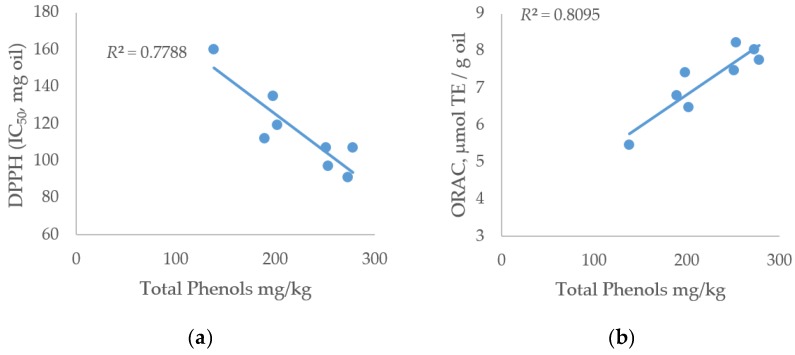
Correlation between total phenolic compounds (mg/kg oil) and antioxidant activity. (**A**) 2,2-Diphenyl-1-picrylhydrazyl (DPPH) assay test (IC_50_, mg oil) and (**B**) oxygen radical absorbance capacity (ORAC) assay test.

The antioxidant activity values were, as expected, closely related to the TPC oil values; Figure 2 shows the correlations between TPC and antioxidant activities. The two antioxidant assays showed a similar correlation coefficient (0.7788 for the DPPH assay and 0.8095 for the ORAC assay).

The measured Pearson correlation coefficient between ORAC and DPPH tests was −0.84, showing a negative correlation between the two tests. This negative value was expected due to the opposite representation of the result (µmol Trolox equivalent/g versus IC_50_ mg oil).

To evaluate the possible correlation between a single compound and antioxidant activity, the Pearson correlation was measured and is reported in Table 6. Decarboxymethyl oleuropein aglycone and decarboxymethyl ligstroside aglycone are the two compounds with a higher correlation to antioxidant activities. In addition, quinic acid shows a high correlation with antioxidant activity, even if the concentration is very low in the analyzed olive oils.

**Figure 3:**
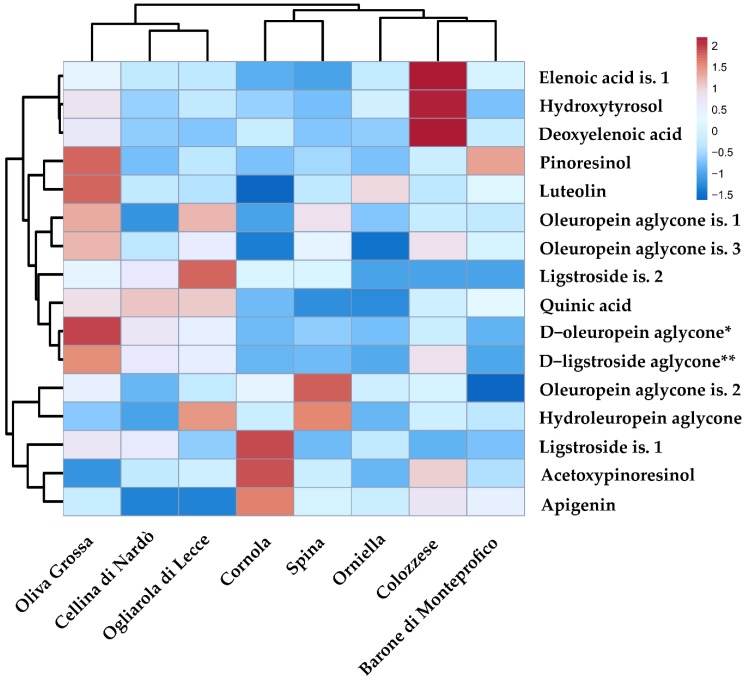
Heatmap showing EVOO chemical compound distribution and concentration among genotypes. A red box means a concentration higher than the mean value among samples. Blue boxes mean lower concentrations. *: decarboxymethyl oleuropein aglycone; **: decarboxymethyl ligstroside aglycone; is.: isomer.

Furthermore, for a better graphical representation of the EVOO chemical compound distribution among genotypes, a heatmap [32] was drawn (Figure 3). The relative unsupervised clustering highlighted two main clusters: a first group including Oliva Grossa, Ogliarola, and Cellina di Nardò genotypes, and a second cluster including Cornola, Orniella, Spina, Colozzese, and Barone di Monteprofico. Among the chemical compounds, decarboxymethyl oleuropein aglycone and decarboxymethyl ligstroside aglycone cluster together.

## 4. Discussion

The TPC detected in the eight monovarietal EVOOs ranged between 138 and 278 mg GAE/kg oil, and they agree with the values reported by Baiano et al. [33] (133–322 mg GAE/kg oil) for olive orchards located in the north of the Apulia region. Similarly, oils from environments like that of the Province of Lecce (Apulia, Italy) showed a TPC of approximately 300 mg GAE/kg oil [10], whereas Ninfali et al. [7] reported values between 50 and 236 mg/kg for plants cultivated in the center of Italy. Ricciutelli et al. [23] analyzed commercial oil and reported values ranging from 136 and 437 mg GAE/kg oil. In Chile [34], Spain [35], Greece [36], and Tunisia [25] the phenolic compounds quantified in olive oil were even higher (680 mg GAE/kg oil). Therefore, the TPC in the eight EVOOs analyzed is in agreement with the average values observed in other extra virgin olive oils [7,10,23]. However, the TPC of EVOOs is strictly related to the olive harvesting time, oil extraction techniques, and also to quantification methodologies [37,38].

Among the many phenolic compounds identified in the eight EVOOs, decarboxymethyl ligstroside aglycone (oleocanthal) deserves special attention because of its several nutraceutical activities highlighted by many studies and reviews [16,20,39,40]. In the analyzed EVOOs, we found high oleocanthal concentrations in Colozzese and Oliva Grossa oils (75.4 and 103.4 mg/kg oil, respectively). The oleocanthal in olive oils showed wide concentration ranges, as reported by many authors [24,26,34,35,41]. In Spanish olive oils, the mean value was 3.90 mg/kg [35], whereas the Sicilian oils had 25 mg/kg, and the Tunisian oils had about 40 mg/kg [41]. Maximum values were reported by Fuentes et al. [34]: 77 mg/kg oil. Considering the central role of this molecule in the nutraceutical properties of EVOO, Colozzese and Oliva Grossa genotypes can represent an excellent source of this compound, and further studies are therefore needed to evaluate the nutraceutical activities of EVOOs produced from the above-mentioned cultivars.

Another interesting molecule present in EVOOs is hydroxytyrosol, which is worthy of investigation for its nutraceutical properties [42]. In the Colozzese cultivar, we found 16 mg of hydroxytyrosol/kg oil, whereas other authors reported much lower values in monovarietal oils, ranging between 0.36 and 2.85 mg/kg oil [33]. In contrast, oils produced from olives in the of south Italy showed a high hydroxytyrosol concentration (41 mg/kg oil) [10], and Ricciutelli et al. [23] have identified a mean value of 9.9 mg/kg oil for high-quality commercial oils. Oil from Spain, Morocco, and Tunisia contain, respectively, 14.34, 6.65, and 22.57 mg/kg oil [24,25,27]. Since EVOO from the Colozzese cultivar also showed a high hydroxytyrosol concentration in comparison with other oils, this cultivar is worthy of interest to obtain EVOO blends with increased nutraceutical properties.

Moreover, oleuropein aglycone, which normally decreases from drupes to malaxation paste and to the final oil [36], ranges from 33.8 to 152.3 mg/kg in the Orniella and Oliva Grossa samples, respectively, and falls within the range of data reported in the literature. In fact, similar values were reported in oils from southern Italy and in oils from Tunisia [10,25,41], whereas lower values were reported in Spanish oils [35]. In contrast, higher values were observed in oils obtained in Morocco [24].

In this report, we performed the analysis of antioxidant activity using two different assays: DPPH and ORAC. Since the solvents of DPPH and ORAC assays are methanol and water, respectively, differences in the reported antioxidant activities are expected. Nevertheless, the tests showed a correlation, as reported by other authors [34,43]. 

Hydroxytyrosol and oleuropein are known as scavengers of hydrophilic peroxyradicals and are usually associated with the antioxidant activities of olive products [44]. However, the data collected from these eight EVOOs did not show a tight correlation between antioxidant activity and the concentration of hydroxytyrosol or oleuropein. As reported by Presti and colleagues [45], hydroxytyrosol is not always correlated to the measured antioxidant activity, whereas the antioxidant activity of oleuropein seems to be linked to the presence of the carboxy-methyl group [6].

The comparison of antioxidant activity between the eight EVOOs and other oils reveals similar trends: Lavelli [46], as well as other authors [10,47], reported DPPH antioxidant activity (IC_50_ values) ranging from 27 to 156 mg. For the ORAC assay, the values ranged from 1.7 and 8.2 μmol TE/g oil in Italian oils [7,48] and from 1.4 and 4.9 μmol TE/g oil in Spanish oils [43]. In the olive oils analyzed in this paper, the ORAC values ranged between 5.4 and 8.0 μmol TE/g oil, high values, on average, for Italian oils. Antioxidant activity is a good index for representing oil quality because it is positively correlated to phenolic compounds (Figure 2 and Table 6).

In particular, we observed that quinic acid, decarboxymethyl oleuropein aglycone, and decarboxymethyl ligstroside aglycone positively correlate to antioxidant activity. However, quinic acid showed low concentrations and probably does not represent the main determinant of the antioxidant properties of these oils. In contrast, decarboxymethyl oleuropein aglycone and decarboxymethyl ligstroside aglycone are known to possess antioxidant activities [6,47,49].

In fact, compounds with o-dihydroxyl functionalities, such as decarboxymethyl oleuropein aglycone, are able to form intramolecular hydrogen bonds during the reaction with free radicals [6,47].

The heatmap plot also highlighted that some compounds are able to characterize some genotypes. For example, elenoic acid, deoxyelenoic acid, and hydroxytyrosol are more concentrated in olive oils from the Colozzese cultivar. In contrast, apigenin, acetoxypinoresinol, and ligstroside is.1 are more concentrated in the Cornola cultivar.

## 5. Conclusions

The results of the present work support the literature in the conclusion that extra virgin oils show a very high variability regarding the content of phenolic compounds. Therefore, a detailed characterization of EVOOs is important to better understand and attest to the antioxidant values and nutraceutical proprieties of this key food of the Mediterranean diet. In addition, the wide variation in the presence of different polyphenols in the tested monovarietal EVOOs demonstrates the remarkable biodiversity typical of Italian olive oils and confirms their nutraceutical and economic value. In particular, Colozzese and Oliva Grossa EVOOs exhibited high amounts of oleocanthal and hydroxytyrosol and good antioxidant activities. Further studies will evaluate nutraceutical features, such as the anti-inflammatory and anti-proliferative activities of such EVOOs, with the aim of suggesting the best use and therefore maximize their health beneficial effects.

## Figures and Tables

**Table 1:** List of olive genotypes used for oil analyses.

Genotype
Colozzese
Barone di Monteprofico
Cellina di Nardò
Cornola
Ogliarola di Lecce
Orniella
Oliva Grossa
Spina

**Table 2:** Total phenolic content (TPC), expressed as mg of gallic acid equivalents (GAE) per kg of oil. Different letters correspond to statistically different means (Duncan’s test, *n* = 3, *p* < 0.05).

Genotype	Total Phenolic Content (mg GAE/kg oil)
Colozzese	251 ± 12 ^a^
Barone di Monteprofico	202 ± 14 ^b^
Cellina di Nardò	253 ± 7 ^a^
Cornola	189 ± 10 ^b^
Ogliarola di Lecce	278 ± 9 ^a^
Orniella	198 ± 3 ^b^
Oliva Grossa	273 ± 3 ^a^
Spina	138 ± 7 ^b^

**Table 3:** High-performance liquid chromatography time-of-flight mass spectrometry (HPLC/MS-TOF) putative identification of the chemical compounds extracted from eight different monovarietal extra virgin olive oils (EVOOs). +: presence; −: absence; Co: Colozzese; Ba: Barone di Monteprofico; Ce: Cellina di Nardò; Cr: Cornola; Og: Ogliarola di Lecce; Or: Orniella; Oo: Oliva Grossa; Sp = Spina.

Peak	Compound	(M-H)^−^	m/z Exp	m/z Calc	Diff. (ppm)	Score	Ref.	Co	Ba	Ce	Cr	Og	Or	Oo	Sp
1	*Quinic acid	C_7_H_11_O_6_	191.0561	191.0563	1.1	94.02	25	+	+	+	+	+	+	+	+
2	*Hydroxytyrosol	C_8_H_9_O_3_	153.0563	153.0557	−2.90	81.59	2, 4	+	+	+	+	+	+	+	+
3	Deoxyelenoic acid	C_11_H_14_O_5_	225.0783	225.0768	−6.36	91.50	2, 25	+	+	+	+	+	+	+	+
4	Hydroxytyrosol acetate	C_10_H_11_O_4_	195.0647	195.0663	8.07	86.46	4	+	+	−	+	+	+	+	+
5	*Vanillic acid	C_8_H_8_O_4_	167.0353	167.0345	3.0	87.23	25	+	−	+	+	+	+	+	−
6	Elenoic acid is. 1	C_11_H_14_O_6_	241.0731	241.0718	−5.71	90.20	2, 25	+	+	+	+	+	+	+	+
7	Tyrosol	C_8_H_10_O_2_	137.0610	137.0605	3.64	87.23	25	+	+	+	+	+	+	+	+
8	*Cumaric acid	C_9_H_15_O_4_	163.0398	163.0401	−3.93	92.50	2, 25	+	−	+	+	+	+	+	+
9	Unknow	−	299.0555	−		−	−	+	−	−	+	+	+	+	+
10	Elenoic acid is. 2	C_11_H_14_O_6_	241.0731	241.0718	−5.71	89.52	2, 4	+	+	+	+	+	+	+	+
11	Deoxyelenoic acid is. 2	C_11_H_14_O_5_	225.0783	225.0768	−6.36	91.50	2, 4	+	−	+	−	+	+	+	+
12	Elenoic acid is. 3	C_11_H_14_O_6_	241.0731	241.0718	−5.71	89.52	2, 4	+	+	−	+	+	+	+	+
13	*Ferulic acid	C_10_H_9_O_4_	193.0507	193.0506	0.51	90.21	4	+	+	+	−	+	+	+	+
14	Decarboxymethyl oleuropein aglycone	C_17_H_19_O_6_	319.1313	319.1187	−7.96	88.20	4, 25	+	+	+	+	+	+	+	+
15	Decarboxymethyl 10-Hydroxy oleuropein aglycone	C_17_H_19_O_7_	335.1163	335.1136	−7.85	87.70	2, 4	+	+	+	−	+	+	+	+
16	Oleuropein aglycone is. 1	C_19_H_21_O_8_	377.1266	377.1242	−6.34	86.70	2	+	+	+	+	+	+	+	+
17	*Pinoresinol	C_20_H_21_O_6_	357.1338	357.1344	1.68	85.32	25	+	+	+	+	+	+	+	+
18	Decarboxymethyl ligstroside aglycone	C_17_H_19_O_5_	303.1252	303.1238	−4.51	90	4	+	+	+	+	+	+	+	+
19	Oleuropein aglycone is. 2	C_19_H_21_O_8_	377.1269	377.1242	−7.09	86.60	2, 4	+	+	+	+	+	+	+	+
20	Hydroxymethyl decarboxymethyl ligstroside aglycone	C_18_H_21_O_6_	333.1360	333.1344	−5	88.69	4	+	+	+	−	+	+	+	+
21	Ligstroside is. 1	C_19_H_21_O_7_	361.1319	361.1351	9.08	87.00	2, 4	+	+	+	+	+	+	+	+
22	*Luteolin	C_15_H_9_O_6_	285.0432	285.0485	−9.78	88.00	2, 4	+	+	+	+	+	+	+	+
23	Acetoxypinoresinol	C_22_H_23_O_8_	415.1404	415.1398	−1.42	98.94	4, 25	+	+	+	+	+	+	+	+
24	Oleuropein aglycone is. 3	C_19_H_21_O_8_	377.1271	377.1242	−7.77	85.60	2	+	+	+	+	+	+	+	+
25	Oleuropein aglycone is. 4	C_19_H_21_O_8_	377.1261	377.1242	−1.90	86.40	2	+	+	+	+	+	+	+	+
26	Ligstroside is. 2	C_19_H_21_O_7_	361.1316	361.1293	−2.30	86.70	2, 4	+	+	+	+	+	+	+	+
27	Methyloleuropein aglycone	C_20_H_23_O_8_	391.1408	391.1398	−2.49	92.63	4	+	+	+	+	+	+	+	−
28	*Apigenin	C_15_H_9_O_5_	269.0459	269.0455	−1.39	86.80	2, 4	+	+	+	+	+	+	+	+
29	Ligstroside is. 3	C_19_H_21_O_7_	361.1321	361.1293	−7.78	86.20	2, 4	+	+	+	+	+	+	+	+
30	Ligstroside is. 4	C_19_H_21_O_7_	361.1321	361.1293	−7.78	86.20	2, 4	+	+	+	+	+	+	+	+
31	10-Hydroxy oleuropein aglycone	C_19_H_21_O_9_	393.1205	393.1191	−3.66	89.95	2	+	+	+	+	+	+	+	+
32	Unknown	−	319.1210	−	−	−	−	+	+	+	+	+	+	+	+
33	Unknown	−	389.2140	−	−	−	−	+	+	+	+	+	+	+	+
34	Unknown	−	346.0352	−	−	−	−	+	+	+	+	+	+	+	+

***m/z Exp:****m*/*z* experimental; ***m/z Calc*** m/z calculated; ***Diff.***: difference between the observed mass and the theoretical mass of the compound (ppm); ***Score***: isotopic abundance distribution match—a measure of the probability that the distribution of isotope abundance ratios calculated for the formula matches the measured data; ***Ref*.**: references; is: isomer. *Confirmed by the authentic chemical standard.

**Table 4:** Quantitative determination of the main chemical compounds present in the extracts from 8 monovarietal EVOOs (mg/kg of oil). Different letters correspond to statistically different means (Duncan’s test, *n* = 3, *p* < 0.05).

Peak	Compound	Colozzese	Barone di Monteprofico	Cellina di Nardò	Cornola	Ogliarola di Lecce	Orniella	Oliva Grossa	Spina
1	Quinic acid	0.6 ± 0.1 ^c^	0.7 ± 0.1 ^b^	1.1 ± 0.1 ^a^	0.2 ± 0.1 ^e^	1.1 ± 0.1 ^a^	0.1 ± 0.1 ^d^	1.0 ± 0.1 ^a^	0.1 ± 0.1 ^d^
2	Hydroxytyrosol	16.1 ± 0.1 ^a^	0.5 ± 0.1 ^d^	1.4 ± 0.2 ^d^	1.37 ± 0.35 ^d^	3.1 ± 0.2 ^c^	4.2 ± 0.7 ^c^	8.8 ± 0.5 ^b^	0.2 ± 0.1 ^d^
3	Deoxyelenoic acid	4.5 ± 0.2 ^a^	1.6 ± 0.4 ^c^	1.1 ± 0.4 ^c^	1.6 ± 0.3 ^c^	1.0 ± 0.1 ^c^	1.1± 0.4 ^c^	2.7 ± 0.2 ^b^	1.0 ± 0.4 ^c^
6	^1^ Elenoic acid is. 1	25.8 ± 0.6 ^a^	9.5 ± 0.8 ^c^	6.9 ± 0.9 ^d^	1.8 ± 0.4 ^e^	6.6 ± 0.8 ^d^	7.1 ± 0.1 ^d^	12.1 ± 0.5 ^b^	0.9 ± 0.5 ^e^
14	^2^ D-oleuropein aglycone*	39.0 ± 0.7 ^d^	7.9 ± 0.1 ^g^	73.6 ± 0.5 ^b^	11.7 ± 0.2 ^f^	64.9 ± 0.6 ^c^	13.2 ± 0.5 ^f^	124.4 ± 0.5 ^a^	19.4 ± 0.3 ^e^
16	^2^ Oleuropein aglycone is. 1	4.2 ± 0.4 ^b^	3.9 ± 0.4 ^b^	0.3 ± 0.4 ^d^	1.0 ± 0.1 ^d^	9.7 ± 0.6 ^a^	2.3 ± 0.3 ^c^	10.1 ± 0.6 ^a^	8.3 ± 0.2 ^a^
17	Pinoresinol	6.5 ± 0.4 ^b^	18.6 ± 0.1 ^b^	1.4 ± 0.4 ^d^	1.6 ± 0.2 ^d^	4.6 ± 0.6 ^bc^	1.6 ± 0.4 ^d^	21.9 ± 0.6 ^a^	3.4 ± 0.5 ^c^
18	^2^ D-ligstroside aglycone**	75.4 ± 0.2 ^b^	4.3 ± 0.2 ^h^	68.3 ± 0.5 ^d^	10.2 ± 0.6 ^f^	64.5 ± 0.8 ^e^	6.7 ± 0.7 ^g^	103.4 ± 0.8 ^a^	12.4 ± 0.8 ^f^
19	Oleuropein aglycone is. 2	28.2 ± 0.7 ^c^	4.3 ± 0.5 ^e^	15.3 ± 0.2 ^d^	33.1 ± 0.7 ^b^	24.1 ± 0.4 ^c^	26.2 ± 0.8 ^c^	34.3 ± 0.8 ^b^	52.6 ± 0.6 ^a^
21	^2^Ligstroside is. 1	2.2 ± 0.5 ^e^	3.3 ± 0.3 ^de^	13.7 ± 0.2 ^b^	23.2 ± 0.6 ^a^	4.2 ± 0.4 ^d^	6.6 ± 0.3 ^c^	14.1 ± 0.3 ^b^	2.7 ± 0.1 ^e^
22	Luteolin	7.6 ± 0.4 ^d^	9.9 ± 0.7 ^c^	8.0 ± 0.7 ^cd^	2.2 ± 0.5 ^e^	7.6 ± 0.3 ^d^	13.2 ± 0.2 ^b^	16.7 ± 0.6 ^a^	7.8 ± 0.5 ^d^
23	^3^ Acetoxypinoresinol	5.6 ± 0.3 ^b^	2.5 ± 0.5 ^c^	2.8 ± 0.8 ^c^	7.4 ± 0.1 ^a^	3.2 ± 0.5 ^c^	1.5 ± 0.5 ^d^	0.8 ± 0.2 ^e^	3.1 ± 0.2 ^c^
24	^2^ Oleuropein aglycone is. 3	93.7 ± 0.4 ^b^	63.6 ± 0.2 ^e^	48.5 ± 0.8 ^f^	8.6 ± 0.1 ^g^	84.6 ± 0.1 ^c^	5.3 ± 0.1 ^h^	107.9 ± 0.7 ^a^	77.5 ± 0.5 ^d^
26	^2^ Ligstroside is. 2	0.8 ± 0.6 ^e^	1.0 ± 0.1 ^e^	31.2 ± 0.7 ^b^	21.4 ± 0.8 ^d^	51.1 ± 0.1 ^a^	1.2 ± 0.1 ^e^	27.1 ± 0.1 ^c^	21.4 ± 0.1 ^d^
28	Apigenin	6.5 ± 0.3 ^b^	5.8 ± 0.6 ^b^	0.2 ± 0.3 ^d^	9.2 ± 0.3 ^a^	0.2 ± 0.1 ^d^	3.9 ± 0.2 ^c^	3.8 ± 0.3 ^c^	4.3 ± 0.4 ^c^
31	^2^10-Hydroxy oleuropein aglycone	53.5 ± 0.8 ^c^	41.3 ± 0.3 ^d^	12.4 ± 0.2 ^g^	52.3 ± 0.9 ^c^	118.3 ± 0.2 ^b^	21.1 ± 0.7 ^e^	29.6 ± 0.8 ^f^	123.0 ± 0.7 ^a^

Determined as: ^1^ Hydroxytyrosol, ^2^ Oleuropein, ^3^ Pinoresinol. *: Decarboxymethyl oleuropein aglycone, **: Decarboxymethyl ligstroside aglycone.

**Table 5:** Antioxidant activity of oil extracts investigated by 2,2-Diphenyl-1-picrylhydrazyl (DPPH) assay (half maximal inhibitory concentration, IC_50_, mg oil) and oxygen radical absorbance capacity (ORAC) assay (μmol Trolox equivalent (TE)/g oil). Different letters correspond to statistically different means (Duncan’s test, *n* = 3, *p* < 0.05).

Genotype	DPPH (IC_50_, mg oil)	ORAC (µmol TE/g oil)
Colozzese	107 ± 8 ^bc^	7.5 ± 0.2 ^b^
Barone di Monteprofico	119 ± 6 ^c^	6.5 ± 0.1 ^c^
Cellina di Nardò	97 ± 4 ^ab^	7.8 ± 0.3 ^b^
Cornola	112 ± 6 ^bc^	6.8 ± 0.1 ^c^
Ogliarola di Lecce	107 ± 7 ^bc^	7.7 ± 0.2 ^b^
Orniella	135 ± 2 ^d^	7.4 ± 0.3 ^b^
Oliva Grossa	91 ± 1 ^a^	8.0 ± 0.1 ^a^
Spina	160 ± 5 ^e^	5.4 ± 0.1 ^d^

**Table 6:** Pearson correlation between antioxidant activities, measured by DPPH and ORAC methods, and EVOO chemical compounds.

Compound	Pearson Correlation Coefficient	
	DPPH Test	ORAC Test
Quinic acid	−0.81	0.65
Hydroxytyrosol	−0.39	0.48
Deoxyelenoic acid	−0.39	0.28
Elenoic acid is. 1	−0.44	0.44
D-oleuropein aglycone*	−0.68	0.69
Oleuropein aglycone is. 1	0.05	0.00
Pinoresinol	−0.35	0.14
D-ligstroside aglycone**	−0.74	0.74
Oleuropein aglycone is. 2	0.49	−0.38
Ligstroside is. 1	−0.44	0.25
Luteolin	−0.13	0.38
Acetoxypinoresinol	−0.02	−0.23
Oleuropein aglycone is. 3	−0.26	0.14
Ligstroside is. 2	−0.31	0.29
Apigenin	0.19	−0.42
Hydroleuropein aglycone	0.49	−0.49

*: Decarboxymethyl oleuropein aglycone, **: Decarboxymethyl ligstroside aglycone.
